# Application of low dose aspirin in pre-eclampsia

**DOI:** 10.3389/fmed.2023.1111371

**Published:** 2023-03-08

**Authors:** Yu Ren, Yong Zhao, Xiangdong Yang, Chaojun Shen, Hua Luo

**Affiliations:** ^1^Department of Pharmacy, Taizhou Hospital of Zhejiang Province Affiliated to Wenzhou Medical University, Taizhou, Zhejiang, China; ^2^Department of Orthopedics, Shanghai Fengxian District Central Hospital, Shanghai, China; ^3^Department of Orthopedics, Ningbo First Hospital, The Affiliated Hospital of Zhejiang University, Ningbo, China; ^4^Department of Orthopedics, Taizhou Hospital of Zhejiang Province Affiliated to Wenzhou Medical University, Taizhou, Zhejiang, China

**Keywords:** aspirin, pre-eclampsia, low-dose, cerebrovascular disease, guideline

## Abstract

Aspirin is widely used in the primary and secondary prevention of cardiovascular and cerebrovascular diseases. Low-dose aspirin is also widely used to prevent pre-eclampsia and fetal growth retardation in utero. However, the use of aspirin during pregnancy is controversial. Since 1985, when aspirin was reported to be effective in obstetrics, numerous studies have attempted to determine the effect of low-dose aspirin on the morbidity of pre-eclampsia but have remained inconclusive. Guidelines for aspirin in preventing pre-eclampsia are different in different countries and regions. This article summarizes the research progress, mechanism, and application prospect of aspirin in preventing pre-eclampsia, providing a theoretical basis for the rational use of aspirin in pregnancy.

## Introduction

Pre-eclampsia (PE), mainly occurring during pregnancy, can lead to severe maternal and fetal complications such as eclampsia, placental abruption, fetal growth restriction, and premature delivery (1). Pre-eclampsia is challenging to diagnose, especially in patients with chronic conditions such as hypertension and proteinuria. Pre-eclampsia can lead to liver and kidney failure, seizures, and abnormalities in the blood clotting system. There is no effective treatment for pre-eclampsia beyond childbirth, making primary and secondary prevention of pre-eclampsia a problematic public health concern. At present, the etiology of PE has not been fully elucidated. There are many theories about its pathogenesis, among which insufficient trophoblast infiltration of spiral arteries and local ischemia and hypoxia of uteroplacental are the mainstream theories. This mechanism involves the vascular system, immune system, inflammatory reaction factors, etc. All factors have mutual influence (2). Clinical studies have shown that early low-dose aspirin (LDA) use in high-risk pregnant women can reduce the risk of early PE (3). Aspirin has been widely used to prevent pre-eclampsia and fetal growth retardation *in utero* (4).

## Research progress of aspirin in prevention of pre-eclampsia

Domestic and foreign guidelines generally recommend LDA in early pregnancy to prevent PE. As early as 1978, it was reported that pregnant women with recurrent gestational hypertension who took 600 mg aspirin daily at 22–32 weeks might be beneficial from gestational hypertension (5). Since then, relevant research has been carried out continuously. In 1979, a retrospective study analyzed the association between aspirin use during pregnancy and the development of pre-eclampsia in primiparas (*n* = 146). It proposed that aspirin use during pregnancy (at least once every 2 weeks) was associated with a reduction in the development of pre-eclampsia (6). In 1985, a study gave 93 pregnant women with high risk factors for pre-eclampsia or fetal growth restriction 300 mg dipyridamole and 150 mg aspirin daily or no medication from the third month of gestation to delivery. Moreover, it was found that early pregnancy medication could prevent pre-eclampsia and fetal growth restriction (7). A meta-analysis in 2001 suggested that low-dose aspirin (60–160 mg) could reduce the risk of pre-eclampsia by 15% in pregnant women with high risk factors for pre-eclampsia without changing the incidence of fetal growth restriction (8). In 2007, another meta-analysis included 59 randomized controlled studies of 37,000 pregnant women at risk for pre-eclampsia (98% were taking aspirin only) to compare the antiplatelet effects in pre-eclampsia, preterm birth (before 34 gestational), and adverse pregnancy outcomes. The results showed that pregnant women at risk for pre-eclampsia who used antiplatelet agents (mainly aspirin) during pregnancy had a 10% reduction in the incidence of pre-eclampsia, preterm birth (before 34 gestation) and adverse pregnancy outcomes (9). The USPSTF Guidelines in 2014 recommended that for women with risk factors (10), LDA (81 mg/ day) for PE prevention was started between 12 and 28 weeks of gestation (preferably before 16 weeks) and continued until delivery. In 2016, a meta-analysis included 6 randomized controlled trials and 898 pregnant women with multiple pregnancies to evaluate the effect of low-dose aspirin in preventing pre-eclampsia and small for gestational age infants. The results showed that low-dose aspirin could prevent the occurrence of pre-eclampsia in multiple pregnancies but had no effect on the incidence of small for gestational age infants (11). A meta-analysis in 2017 of 45 randomized controlled studies involving 20,909 high-risk pre-eclampsia women showed the incidence of pre-eclampsia, severe pre-eclampsia, and fetal growth restriction was compared between pregnant women taking 50–150 mg aspirin daily at ≤ 16 or >16 weeks of gestation. Moreover, for those taking or not taking a placebo, it was proposed that aspirin use in early pregnancy (≤ 16 weeks of gestation) can prevent pre-eclampsia and fetal growth restriction (3). In 2017, the largest multicenter, double-blind, randomized controlled trial in Europe randomly assigned 1,776 singleton pregnant women > 18 years of age with high risk factors for preterm pre-eclampsia (<37 weeks of gestation) to aspirin or placebo in a 1:1 ratio. The incidence of preterm pre-eclampsia and adverse neonatal outcomes (e.g., stillbirth, neonatal death, and the need for intensive care or positive pressure ventilation) was compared between 11–14 and 36 weeks of gestation with 150 mg aspirin or placebo every night. The incidence of preterm pre-eclampsia was found to be lower in the low-dose aspirin group (1.6%) than in the placebo group (4.3%) (12). Based on these studies, the ISSHP guidelines in 2018 recommended that people at high risk of pre-eclampsia (as shown in Table 1) can take low-dose aspirin (75–162 mg/d) before 16 weeks of pregnancy to prevent pre-eclampsia (13). In 2019, the ACOG guidelines updated pre-eclampsia prevention indications, pre-eclampsia risk factors were divided into high risk and intermediate risk factors. This guideline recommends that pregnant women with one or more high risk factors for pre-eclampsia or two or more moderate risk factors for pre-eclampsia should take low-dose aspirin (81 mg/d) from 12 to 28 weeks of gestation (preferably before 16 weeks of gestation) until delivery (14). The summary table with criteria and doses of aspirin for prevention of international preeclampsia guidelines was shown in Table 1.

**Table 1:** Aspirin criteria and doses for prevention of preeclampsia.

**Year and committee**	**Source**	**Population**	**Recommendation**	**Grade**
2014 USPSTF (10)	Low-dose aspirin use for the prevention of morbidity and mortality from preeclampsia: U.S. preventive services task force recommendation statement	Asymptomatic pregnant women who are at high risk for preeclampsia	Prescribe low-dose (81 mg/d) aspirin after 12 weeks of gestation.	B
2018 ISSHP (13)	The hypertensive disorders of pregnancy: ISSHP classification, diagnosis & management recommendations for international practice	Pregnant women at increased risk for preeclampsia	Aspirin should be given at a dose between 100 and 150 mg per day, started preferably before 16 weeks' gestation, possibly taken at night, and continued until delivery.	/
2019 ACOG (14)	Gestational hypertension and preeclampsia	Women with any of the high-risk factors for preeclampsia and those with more than one of the moderate-risk factors	Those should receive low-dose (81 mg/day) aspirin for preeclampsia prophylaxis, initiated between 12 and 28 weeks of gestation (optimally before 16 weeks of gestation) and continuing until delivery.	A
2019 FIGO (15)	The international federation of gynecology and obstetrics (FIGO) initiative on pre-eclampsia: A pragmatic guide for first-trimester screening and prevention	Women identified at high risk	Receive aspirin prophylaxis commencing at 11–14 + 6 weeks of gestation at a dose of ~150 mg to be taken every night until either 36 weeks of gestation, when delivery occurs, or when PE is diagnosed.	/
2019 NICE (16)	Hypertension in pregnancy: diagnosis and management	Pregnant women at high risk or with more than 1 moderate risk factor for preeclampsia	Take 75–150 mg of Aspirin daily from 12 weeks until the birth of the baby.	/
2021 IAPM (17)	International Academy of Perinatal Medicine (IAPM) guidelines for screening, prediction, prevention and management of pre-eclampsia to reduce maternal mortality in developing countries	Women at preeclampsia risk	150 mg of aspirin daily at bed time starting from 11 to 14 weeks of pregnancy up until 36 weeks.	/
2021 ISSHP (18)	The 2021 International Society for the Study of Hypertension in Pregnancy classification, diagnosis and management recommendations for international practice	Women at increased risk of pre-eclampsia	Low-dose aspirin is recommended to be taken at bedtime, preferably before 16 weeks and discontinued by 36 weeks. After multivariable screening, aspirin should be given at a dose of 150 mg/night. After screening with clinical risk factors and BP, aspirin should be given at a dose of 100–162 mg/d.	Strong
2021 WHO (19)	WHO recommendations on antiplatelet agents for the prevention of pre-eclampsia	Pre-eclampsia in women at moderate or high risk of developing the condition.	Low-dose acetylsalicylic acid (aspirin, 75 mg per day) is recommended. Low-dose acetylsalicylic acid (aspirin, 75 mg per day) for the prevention of pre-eclampsia and its related complications should be initiated by 20 weeks' gestation or as soon as antenatal care is started.	/

## Mechanism of aspirin use in pre-eclampsia

Due to the complex etiology of PE, the treatment effect is poor, and they even have to terminate the pregnancy. However, even after the termination of pregnancy, some patients are still accompanied by chronic hypertension or other cardiovascular diseases for a long time (20). Therefore, clarifying the specific pathogenesis of PE is of great significance for preventing and treating the disease. The onset of PE had previously been considered with shallow placenta implantation, genetic predisposition, excessive inflammation, endothelial dysfunction, the disorder of maternal-fetal immune balance, and other factors, including uterine placenta perfusion pressure decrease. Resulting in the placenta drawing more attention, the mechanism of ischemia hypoxia may increase the body and the placenta's local release of bioactive factors, and extensive vascular dysfunction ensues and leads to hypertension.

Studies have shown that aspirin prevents pre-eclampsia under strict indications (21, 22). The degree of platelet activation in pregnant women with pre-eclampsia is significantly higher than that in ordinary pregnant women, manifested by shortened APTT and hypercoagulable state (23). Aspirin can regulate the homeostasis of thromboxane and prostacyclin. Moreover, it also inhibits platelet aggregation, which can prevent the formation of small thrombosis, reduce the organ function damage caused by thrombosis attachment, and play a role in preventing pre-eclampsia.

### Aspirin reduces TXA2/PGI2

**Figure 1:**
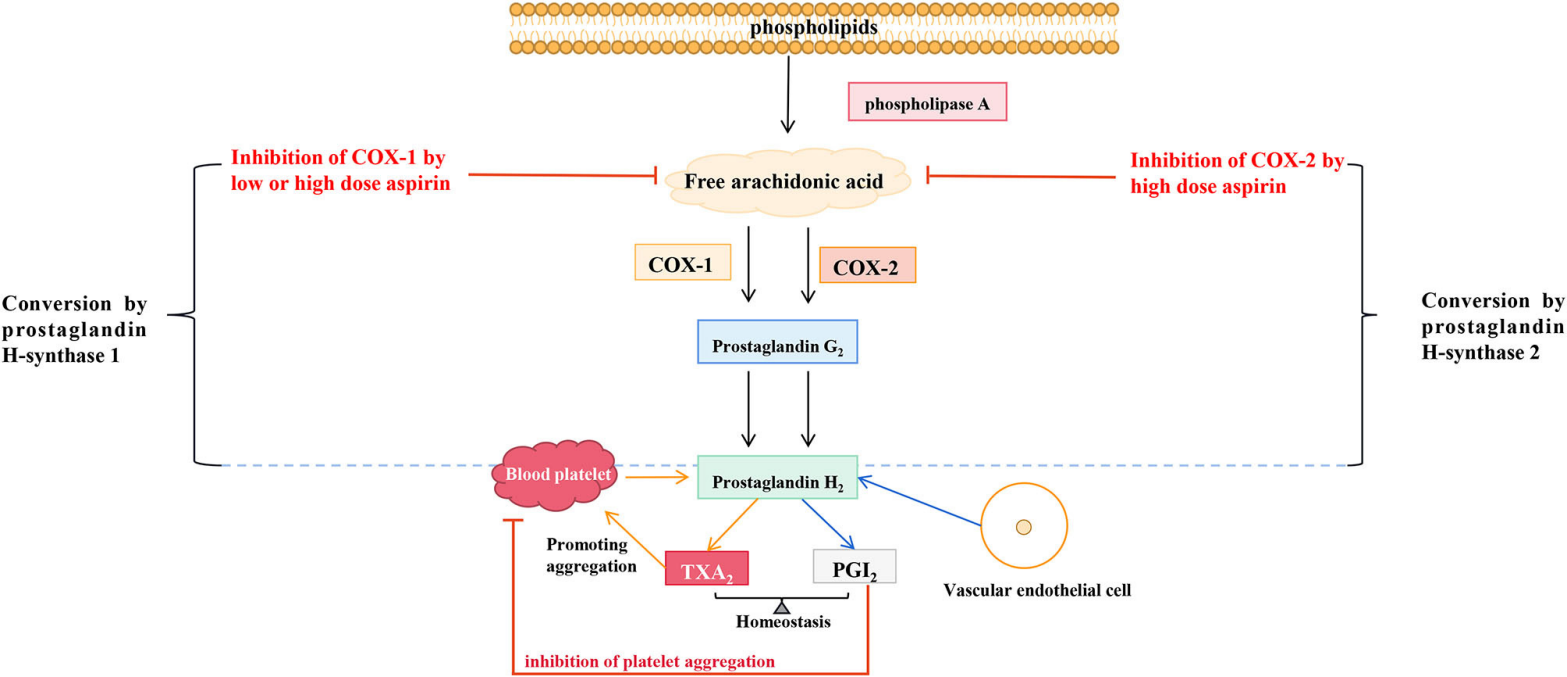
The *in-vivo* mechanism of aspirin in endothelium and blood vessels.

TXA2 constricts blood vessels, promotes platelet aggregation, and induces thrombosis, and PGI2 is the most effective endogenous inhibitor of platelet aggregation. COX activation and prostacyclin synthetase inhibition in pre-eclampsia produce a rapid imbalance of TXA_2_/PGI_2_, leading to clinical symptoms. LDA has been reported to reverse the TXA_2_/PGI_2_ imbalance. Its mechanism is that it can inhibit the secretion of TXA_2_, lead to the reduction of platelet aggregation, and does not change the secretion of PGI_2_ by endothelial cells. Hence, it is conducive to relaxing blood vessels and reducing blood pressure (24). Aspirin has long been used in treating cardiovascular and cerebrovascular diseases and acts by regulating the production of TXA_2_ and PGI_2_. As early as 1983, a study proposed that long-term high doses of aspirin (20–2,600 mg/d) in healthy male volunteers could inhibit the biosynthesis of endogenous TXA_2_ and PGI_2_, and aspirin dose over 80 mg/d could significantly inhibit the biosynthesis of endogenous PGI_2_ (25). A study of 40 pregnant women (36 weeks + 5 to 37 weeks + 2 of gestation) randomly assigned to receive a placebo, 20, 60, or 80 mg aspirin daily until delivery showed that after 1 week of treatment, TXA_2_ production was reduced in more than 90% of women in the 60 mg and 80 mg aspirin groups. However, aspirin dose did not affect the serum levels of 6-keto-prostaglandin F1α and thromboxane B2 in neonates (24). Daily LDA significantly reduces the incidence of gestational hypertension and pre-eclampsia by reducing TXA_2_/PGI_2_ (26, 27). LDA preferentially inhibits TXA_2_ synthesis and restores the imbalance between TXA_2_ elevation and PGI_2_ reduction in pre-eclampsia (28). Therefore, aspirin mainly inhibits COX-1, reduces TXA_2_ synthesis, reduces TXA_2_/PGI_2_, and avoids the occurrence of pre-eclampsia. However, the mechanism by which aspirin selectively inhibits COX-1 remains unknown and requires further investigation. The *in-vivo* mechanism of aspirin in endothelium and blood vessels was shown in Figure 1.

### Aspirin reduces sFlt-1 production

In patients with pre-eclampsia, sFlt-1 is expressed at a high level *in vivo*, which undoubtedly leads to the imbalance of angiogenesis and is one of the main pathogeneses of pre-eclampsia. Reducing sFlt-1 production is a promising method to prevent and treat pre-eclampsia. The possible mechanism of aspirin reducing sFlt-1 is the inhibition of COX-1 and amino-terminal kinase JNK/AP-1 pathway. Primary cytotrophoblast cells from normal placenta were treated with aspirin or COX-2 inhibitor celecoxib. Then sFlt-1 expression was measured. Meanwhile, trophoblasts in the first trimester (HTR-8/SVneo cells) were co-cultured with aspirin. It was found that aspirin inhibited the production of sFlt-1 in cytotrophoblast cells and HTR-8/SVneo cells. The effect of COX-1 inhibitor sc-560 on the expression and release of sFlt-1 in cytotrophoblast cells was similar to that of aspirin, suggesting that aspirin may play a role by inhibiting COX-1 (29). When human choriocarcinoma cells (JEG3 cells) were exposed to hypoxia, the phosphorylation levels of JNK, c-Jun, and c-Fos genes were increased, which was prevented by aspirin. After overexpression or knockdown of c-Jun and c-Fos, the level of sFlt-1 in JEG3 cells was increased or decreased, respectively. Subsequently, luciferase reporter gene analysis showed that low-dose aspirin could directly reduce the expression of transcription factors in the upstream promoter region of sFlt-1, thereby reducing the production of sFlt-1 (30). These results suggest that aspirin attenuates hypoxia-induced activation of the JNK/AP-1 pathway in trophoblast cells, thereby inhibiting sFlt-1 production. In conclusion, aspirin can inhibit hypoxia-induced sFlt-1 production by inhibiting COX-1 and JNK/AP-1 and ultimately prevent pre-eclampsia. However, whether these two mechanisms are the primary mechanism by which aspirin reduces sFlt-1 production, which one plays the leading role, and whether other mechanisms reduce sFlt-1 remains unknown.

### Aspirin increases the proliferation and invasion of trophoblast cells

Trophoblast invasion is essential in placenta growth, maturation, and +0-normal pregnancy. Impaired trophoblast invasion is one of the pathogeneses of pre-eclampsia. Therefore, researchers have conducted relevant studies on the effect of aspirin on trophoblast cells and believe that the effect of aspirin on trophoblast cells is mainly to increase the proliferation and invasion of trophoblast cells and inhibit the apoptosis of trophoblast cells. In an *in vitro* study, JEG3 cells and human umbilical vein endothelial cells were exposed to hypoxia (2%) or normoxia (21%), and it was found that the addition of low-dose aspirin in culture could inhibit hypoxia-induced trophoblast apoptosis and promote trophoblast migration and invasion. However, the specific mechanism was unclear (30). Xu et al. (31) showed that TNF-α could inhibit trophoblast cells integrated into the endothelial cell network. However, aspirin could inhibit this effect, increase trophoblast cytokine release, reduce cell apoptosis, change cell aggregation and fusion, and ultimately improve the function of trophoblast cells (32).

### Anti-inflammatory effects

Excessive activation of inflammatory immunity and damage of vascular endothelial cells are also essential mechanisms of the pathogenesis of pre-eclampsia. Therefore, active anti-inflammatory makes sense in the prevention of pre-eclampsia. Sun et al. (33) found that aspirin could improve the PE symptoms induced by lipopolysaccharide by inhibiting the release of inflammatory factors in the placenta and serum. The anti-inflammatory mechanism of aspirin may be the inhibition of microRNA (miR)-155/ nitric oxide synthase axis and the reduction of inflammatory factor production. Studies have found that aspirin inhibits the expression of the nuclear factor-κB-dependent miR-155 host gene, increases the expression of nitric oxide synthase, and induces the release of nitric oxide, thereby producing vasorelaxation and anti-inflammatory effects (34). A study isolated, cultured, and identified decidual mesenchymal stem cells from full-term placentas from ordinary and pre-eclamptic women and treated them with aspirin. Aspirin reduced the production of inflammatory cytokines (interferon-γ and interleukin-8). Enhanced antioxidant capacity (increased activities of superoxide dismutase, catalase, and glutathione peroxidase) and adhesion of human decidual mesenchymal stem cells in term pre-eclampsia (33). Animal experiments have confirmed that aspirin may also exert anti-inflammatory effects by inhibiting NF-κB p65/TLRs/MyD88 signaling pathway and reducing the production of interleukin-6, 1β, tumor necrosis factor-α and interferon-γ (35). Therefore, aspirin may avoid the occurrence of pre-eclampsia by exerting its anti-inflammatory effect, but the mechanism by which it inhibits the production of inflammatory factors still needs to be further studied.

In a word, because the pathogenesis of pre-eclampsia is relatively complex, aspirin to prevent pre-eclampsia is the outcome of the combined action of multiple mechanisms. Thus, further studies are still needed to elucidate the underlying mechanism, providing theoretical for pre-eclampsia prevention and treatment and new diagnostic and therapeutic targets for pre-eclampsia, eventually for clinical services.

## Aspirin dosage and moment of administration

The starting time and dose of oral aspirin are controversial. A meta-analysis of 32,217 subjects by Meher et al. (36) showed that aspirin could reduce the risk of PE by 10%, and there was no difference in the effect of oral aspirin before and after 16 weeks of pregnancy on the risk of PE. However, the study of Meher et al. (36) has defects in methodology and understanding of the perinatal disease mechanism. Roberge et al. (3) included 45 randomized controlled studies (RCTS) with 20,909 subjects in their meta-analysis. They found that aspirin starting at ≤ 16 gestational weeks could effectively reduce the risk of PE (RR = 0.57), and the effect of daily oral aspirin ≥ 100 mg was much more significant. Moreover, the effect of preventing preterm PE is better than that of PE. Seidler et al. (37) believed that Roberge et al. (3) had limitations in their study on dose effect. They could not control the study effect, maternal risk factors, and publication bias. This study included 22 RCTs with a total of 30,532 subjects based on individual patient information, including 13 studies with a daily aspirin dose ≤ 81 mg and 9 studies with a daily aspirin dose > 81 mg. The risk of recurrent PE and preterm PE at different doses was compared. The results of the two studies were compared with the ASPRE study (oral aspirin 150 mg daily) (22), and it was found that the daily dose of aspirin ≤ 81 mg could reduce the risk of PE (RR = 0.92), and the effect was more noticeable when the dose was > 81 mg (RR = 0.74). The dose of 150 mg was more effective than the former (RR = 0.72). The effect of preventing preterm PE was more significant than that of PE (≤ 81 mg, RR = 0.86; >81 mg, RR = 0.75; 150 mg, RR = 0.38). Roberge et al. (38) conducted a meta-analysis of 12,585 subjects. They found that aspirin did not increase the risk of prenatal bleeding and placental abruption. When aspirin was ≥100 mg daily, it was safer to start taking aspirin at ≤ 16 gestational weeks. More data are needed before aspirin can be used for pre-eclampsia prevention, or national guidelines can be modified.

## The problem for aspirin applied to pre-eclampsia

However, with the deepening of studies on aspirin prevention of thrombotic diseases, it was found that some patients still could not achieve the desired effect after receiving standardized aspirin treatment, which is called aspirin resistance (AR) (39, 40). Other antiplatelet agents, like clopidogrel, may improve in patients who do not respond to aspirin. Presently, the definition of AR has yet to be unified, divided into two types–clinical resistance and laboratory resistance (41). Clinical resistance is when aspirin does not prevent ischemic events as intended; Laboratory resistance means that aspirin does not effectively inhibit platelet aggregation as measured by platelet activity *in vitro*. Epidemiological studies have shown that 1/3 of the occurrence of AR is determined by genes (42). Genetic variation of genes involved in various proteins encoding the mechanism of aspirin may lead to differences in the concentration of active drugs, thus leading to AR (39). Wójtowicz et al. (43) found that the incidence of minor for gestational age infant (SGA), fetal distress, and pre-eclampsia in the AR group was higher than that in the aspirin-sensitive group (AS group), and the differences were statistically significant (*P* = 0.003, 0.014, and 0.003, respectively). Therefore, pregnant women who do not respond to aspirin are at higher risk for adverse pregnancy outcomes such as pre-eclampsia and fetal growth restriction.

The correlation between gene polymorphism and aspirin resistance has become a research hotspot. The polymorphisms of ABC transporter family genes, cyclooxygenase (COX) genes (COX-1 and COX-2), TXA2R gene, ADP receptor gene, and GP receptor gene and their interactions have been widely studied. Since it has rarely been studied in pregnant women, more data are needed before aspirin can be used for pre-eclampsia prevention or before national guidelines are changed. To guide the individualized use of aspirin in high-risk obstetric populations and improve pregnancy outcomes.

## Conclusion

Because the pathogenesis of pre-eclampsia is complicated, aspirin prevention results from multiple mechanisms. Based on these reports, it can be concluded that low-dose aspirin treatment is effective in the secondary prevention of pre-eclampsia in high-risk patients. The indications for aspirin for primary prevention are controversial, and recent reports suggest strategies to recommend aspirin for high-risk patients. The usefulness of this strategy is still being evaluated, and more data are needed before practical implementation. Aspirin should be taken daily in the evening at doses ranging from 80 to 150 mg. Evidence suggests that aspirin's efficacy has a dose effect, increasing with the dose. However, the potential fetal toxicity of aspirin should not be ignored because it can cross the placental barrier and inhibit fetal platelet aggregation. However, LDA has a high safety profile and is considered to have no apparent toxicity to the mother or fetus. The number of patients exposed to doses > 100 mg is low, and the safety of a prophylactic strategy based on 150 mg aspirin per day must be confirmed. Gene polymorphisms related to aspirin metabolism and action are related to individual responses to aspirin. Genetic testing may provide a new basis for selecting antiplatelet aggregation drugs for preventive treatment of pre-eclampsia to achieve individualized precision medicine.

## Author contributions

HL and YR conceived and designed the project. CS performed the literature retrieval. YR drafted the article. XY and YZ conceived the project and provided suggestions to improve it. HL developed the idea for the study and finally revised the paper. All authors contributed to the article and approved the submitted version.
